# Tracking visual outcomes – Follow‐up on patients born preterm with childhood‐onset visual impairment

**DOI:** 10.1111/aos.17461

**Published:** 2025-02-15

**Authors:** Hajer A. Al‐Abaiji, Kamilla Rothe Nissen, Carina Slidsborg, Morten La Cour, Line Kessel

**Affiliations:** ^1^ Department of Ophthalmology Copenhagen University Hospital – Rigshospitalet Copenhagen Denmark; ^2^ Department of Clinical Medicine University of Copenhagen Copenhagen Denmark

**Keywords:** health‐related quality of life, prematurity, retinopathy of prematurity, visual impairment

## Abstract

**Purpose:**

Preterm birth is associated with a risk of ocular complications. The primary aim of the study was to evaluate the main cause of visual impairment (VI) in a cohort of preterm born patients who had childhood‐onset VI. The association between health‐related quality of life (HRQoL) and age, cause—and severity of VI and impairment type was assessed.

**Methods:**

In Denmark, patients <18 years with VI are enrolled at the National Danish Registry of Children with Visual Impairment (NDRCVI). Patients born preterm and enrolled at NDRCVI at any time between 1988 and 2020 were invited for a follow‐up regardless of current age. Examinations performed were visual acuity (VA), refraction, Goldmann visual fields, ocular biometry and posterior segment imaging. The three main outcomes were VA sorted by VI severity (mild, moderate, severe and blind), a score for HRQoL (HUI3) and the main cause of VI.

**Results:**

Thirty‐two preterm‐born patients participated in the study with a median age of 28 years at examination (range 8–43). ROP was the main cause of VI in 15 patients (47%) followed by CVI in eight patients (25%). Increasing age at assessment had a significantly negative impact on the HRQoL‐score, when unadjusted for type of impairments. HRQoL was significantly lower in patients with combined impairments compared to isolated VI (*p* = 0.003).

**Conclusion:**

ROP and CVI were the most common causes of VI. The HRQoL was significantly lower when VI was combined with other impairments in contrast to isolated VI. Reducing cerebral damage in preterm born children is likely to enhance both HRQoL and visual function.

## 1 INTRODUCTION

Patients surviving prematurity are in risk of complications that follow them into adulthood. Several studies have investigated the long‐term ophthalmologic and neurologic outcomes in patients born preterm, indicating that follow‐up is crucial to manage the life‐long complications (Jain et al., 2022; Mitha et al., 2024). Both moderate and very preterm birth are associated with a spectrum of impairments affecting multiple organ systems due to the immature state of these infants at birth. These can include reduced cognitive abilities and psychomotor impairments, which collectively may contribute to a complex health profile (Mitha et al., 2024; Pascal et al., 2018). One thing is managing such complications, but it is also very important to address how they impact the health‐related quality of life (HRQoL).

In Denmark, patients with visual impairment (VI) are enrolled at the National Danish Registry of Children with Visual Impairment (NDRCVI) (Kessel et al., 2024). The registry contains information about gestational age (GA), birth weight (BW), ophthalmic status including ophthalmic diagnoses, severity of VI, the main cause of VI and impairments beyond the VI. Information in the registry is continuously updated based on copies of medical files that are sent to the registry from treating physicians as long as the child is enrolled in the register.

In children born preterm, three primary causes of VI should be addressed. First, retinopathy of prematurity (ROP) a sight‐threatening eye disease resulting from disrupted retinal maturation in preterm infants (ICROP, 2021). Studies indicate that reduced brain volume is associated with all stages of ROP, suggesting a shared pathway of impairment affecting both the neurovascular‐ and neural development (Sharma et al., 2022; Sveinsdóttir et al., 2018) This association underscores that cerebral damage itself can affect the visual function in preterm children, with and without ROP (Hellström et al., 2022; Holmström et al., 2014; Slidsborg et al., 2012) Second, cerebral visual impairment (CVI) a common cause of VI in preterm children with low GA, is often associated with other neurological diseases such as cerebral palsy and intellectual disability (Dutton, 2013; Sakki et al., 2021). This type of injury can cause visual field defects and subnormal visual acuity, strabismus, nystagmus, ocular motor problems and cognitive‐perceptual visual impairment (Merabet et al., 2017; Sakki et al., 2021). The main challenge in managing CVI is the lack of early diagnosis, exacerbated by the absence of national guidelines for diagnosing CVI in Denmark. However, while international guidelines have been introduced recently, they have not yet been practically implemented in Denmark (Boonstra et al., 2022; Kessel et al., 2024). Third, optic nerve atrophy (ONA) often results from damage to the brain's visual pathways, leading to degeneration of retinal ganglion cells. Advanced imaging techniques have confirmed the link between brain damage and ONA (Sharma et al., 2022). Additionally, an excavated optic disc has been reported to be more evident in patients with CVI (Ruberto et al., 2006).

The present study was conducted to investigate the association between HRQoL and age, cause‐ and severity of VI and type of impairment seen in preterm born patients. Previous studies have shown reduced HRQoL in patients with CVI and patients with impairments (Bolbocean et al., 2023; Sakki et al., 2021). However, the possible co‐occurrence of CVI and multiple impairments in preterm born patients, made us hypothesize that patients born preterm with childhood‐onset VI might experience a considerable effect on their HRQoL. It is crucial to address the risk factors impacting HRQoL in this population to improve rehabilitation services and provide appropriate guidance to caregivers of preterm infants. Furthermore, we aimed to investigate whether the ocular condition and visual function had changed since the last entries in the registry.

## 2 METHODS

### 2.1 The National Danish Register of Children with Visual Impairment (NDRCVI)

The NDRCVI is a national register of children with VI. To be enrolled in the registry, the child must be <18 years and have a best corrected VA ≤20/60. In addition, children with severe visual field defects, i.e., hemianopia or visual field <20°, or genetic, progressive, retinal diseases are enrolled irrespective of visual function. Furthermore, children may be enrolled even if their visual function cannot be measured or if VA is >20/60 but the functional use of the vision equivalates VI, e.g., in case of severe cerebral visual impairment (CVI). If the vision improves >20/60 during childhood or when the patient turns 18, they are deregistered from the NDRCVI. A register analysis of the entire preterm cohort enrolled at NDRCVI from 1988 to 2020 was investigated in a previous paper (Al‐Abaiji et al., 2024) In this study, a subgroup of the register cohort was invited for a follow‐up examination.

### 2.2 Population and inclusion

The study was a cross‐sectional study of a subgroup of patients born prematurely with childhood‐onset VI enrolled at NDRCVI. We included patients born at gestational age (GA) <32 weeks who had been registered at NDRCVI at any time between 1988 and 2020 regardless of the present age. We excluded patients who were deregistered from the NDRCVI due to improved vision before the age of 18. Patients with implausible birth parameters, e.g., gestational age of 23 weeks and a birth weight of 2000 g, were also excluded from the study. Furthermore, patients had to live within a reasonable geographical distance from the hospital in order to complete the examinations within 1 day. Thus, patients residing in the Regions of Central and Northern Jutland were not invited.

Patients residing in Capital Region of Denmark, Region Zealand and Southern Denmark Region were invited by digital letter twice and in case of non‐response they received a telephone call. In addition to a written information leaflet patients were also informed of the study by their vision therapists.

### 2.3 Eye examination

Subjective refraction was measured using the Early Treatment Diabetic Retinopathy Study charts (ETDRS chart R, 4‐m original series, Precision‐Vision, La Salle, IL) with the objective refraction measured with the Retinomax (Righton, Tokyo, Japan) or by retinoscopy as a starting point. Final refraction was determined after dilating eye drops using cyclopentolate 1% administered twice in five‐minute intervals or pupil dilation was obtained using tropicamide 0.5% and phenylephrine 2.5% in pseudophakic or aphakic younger patients. Otherwise tropicamide 1% and phenylephrine 10% was used for adults. The final refraction is presented in spherical equivalent calculated by the following equation:

$$\text{Spherical equivalent}=\text{spherical power}+\left(\frac{\text{Cylinder power}}{2}\right)$$

VA was measured using the ETDRS charts 1 and 2 for the right and left eye, respectively (ETDRS, 4‐m original series, Precision‐Vision, La Salle, IL). VA was assessed using preferential‐looking tests for non‐verbal patients and VA was reported in feet using the numbers provided on the backside of the cards depending on the distance at which the cards were used. VI was grouped based on severity into mild VI (VA >20/60, logMAR <0.5), moderate VI (VA ≤20/60 to >20/200, logMAR 0.6 to 1), severe VI (VA ≤20/200 to >20/1250, logMAR 1.1 to 1.30) and blind (VA ≤20/1250 to no light perception (–LP), logMAR >1.40), based on the better seeing eye. The visual fields were evaluated using Goldmann kinetic perimetry target size III4e. Visual fields are reported in total sum of the extent of eight meridians (15°, 60°, 105°, 150°, 195°, 240°, 285° and 330°) using the ImageJ program by converting pixels to degrees (Larsson et al., 2004; McLoone et al., 2007) A visual field defect of ≤20° or hemianopsia would lead to a one level downgrade in VI severity group and visual field ≤10° would group the patient as blind, regardless of the measured VA.

Ocular biometry was obtained using swept‐source optical coherence interferometry biometer IOL Master 700 (Carl Zeiss Meditec AG, Germany) and is reported as lens thickness (LT), anterior chamber depth (ACD), central cornea thickness (CCT) and axial length (AL). Anterior segment OCT (CASIA2, Tomey, Nagoya, Japan) was used to image and measure the anterior segment if IOL master could not be performed due to band keratopathy or nystagmus. Corneal curvature was evaluated using Retinomax K‐plus 3 (Bon, Tokyo, Japan). Intraocular pressure (IOP) was measured using rebound tonometry (Icare ic100, Icare Oy, Finland). Wide field fundus imaging using Clarus 500 (Carl Zeiss Meditec, Jena, Germany) was performed after pupil dilation to visualize the center and periphery of the retina by merging a set of images.

The presetting of a fast macular volume scan (20° × 20°), macular single scan (100 frames) and circular retinal nerve fibre layer scan (100 frames) around the optic disc was performed using Spectral‐domain optical coherence tomography (SD‐OCT, Spectralis; Heidelberg Engineering, Heidelberg, Germany).

Finally, an ophthalmological examination of the anterior and posterior segments was performed by an experienced paediatric ophthalmologist (KRN) using slit lamp and indirect ophthalmoscopy. The optic disc was evaluated using the most specific technique it was possible to use, e.g., fundus photographs and OCT were used whenever possible but when not possible it was diagnosed by fundoscopy. No direct diagnosis of CVI was made through the review of CT, MRI or neuropsychological assessments. Therefore, if the level of VI could not be explained by any ocular findings or damage to the anterior visual pathways, these patients were strongly suspected of having CVI and were categorized accordingly (Sakki et al., 2018, 2021).

For patients with severe band keratopathy or patients who could not corporate, an ultrasound examination was performed. In case of several concomitant eye diseases, the ophthalmologist (KRN) decided the main cause of VI based on the ocular condition with the greatest effect on VA during the examination.

HRQoL was evaluated with the Health Utility Index 3 questionnaire (HUI 3, Canada) (Horsman et al., 2003). We used two versions of the HUI 3 depending on the age and capability of the patient. If the patient was young or intellectually disabled, the first version was used and filled out by a guardian on behalf of the patient. The second version was self‐administered and used if the patient was an intellectually capable adult. A mean HRQoL score was calculated for each patient with a validated algorithm for the multi‐attribute utility (Feeny et al., 2002; Feng et al., 2009). The expected value interval spans from – 0.36 to 1.00, where 0 is equivalent to dead and 1.0 is equivalent to the best health state. A negative value was defined as a status worse than dead (Feeny et al., 2002; Horsman et al., 2003). The score was finally labelled in disability severities as none (>0.99), mild (0.89–0.99), moderate (0.70–0.88) and severe (<0.70) (Feng et al., 2009). A single score for each of the eight attributes (vision, hearing, speech, ambulation, dexterity, emotion and cognition) of the questionnaire was also calculated.

### 2.4 Data access and management

From the NDRCVI, we extracted place of residency, year at enrollment, main cause of VI, VI severity and type of impairment. Type of impairment was either isolated VI or VI combined with other impairments, such as mental retardation, physical disability, hearing loss or psychomotor impairment (labelled combined impairments in the rest of the manuscript). The medical files were reviewed for the subgroup of patients who were invited and gave informed consent to participate in the cross‐sectional study. This included a review of birth weight, gestational age and neonatal medical history. Data were collected and managed using REDcap (Research Electronic Data Capture, Vanderbilt University, US).

The statistical and descriptive analysis was performed using STATA version 18. Nonparametric categorical variables were analysed with Mann–Whitney, and continuous variables were analysed with an unpaired *t*‐test when comparing non‐attendees with attendees. Chi‐square test was used to analyse a potential difference in the distribution of the VI severity groups among (categorical variables). The main cause of VI and change VI severity is presented per patient. The association between HRQoL and age and BW was analysed with Spearman's correlation. A regression analysis was performed to evaluate the effect on HRQoL by age and type of impairment. A *p*‐value below 0.05 was considered statistically significant.

### 2.5 Approvals

This study followed the tenets of the Helsinki declaration. Access to the information from the NDRCVI was approved by institutional review. The cross‐sectional part of the study was approved by The Danish National Committee on Health Research Ethics (VEK. Jr. H‐21013557) as well as by the Danish Data Protection Agency (P‐2019‐288). Patients participating in the cross‐sectional study were given oral and written information and the patients signed a consent form prior to participation. Four versions of the informed consent were prepared according to the rules of the Ethical committee in Denmark (1) one for children under 17 years, where both parents gave consent, (2) one for adolescents between 17 and 18, (3) one for adults aged 18+ and lastly and (4) one for intellectually disabled patients accompanied by a guardian.

## 3 RESULTS

### 3.1 Population

**FIGURE 1:**
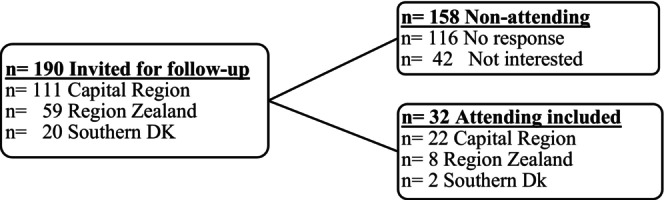
Flow‐chart. The invited patients and the patients accepting the invitation.

We invited 190 patients born <GA 32 who had childhood‐onset VI, of whom 32 (17%) agreed to participate (Figure 1). The patients attending were not statistically different from non‐attendees by sex, GA, BW, comorbidities or VI severity (Table 1). The attending patients had a median age of 28 years (IQR: 17–32, range: 8–43). Ten (31%) were still enrolled in the registry (<18 years). The duration of follow‐up from latest entry in the registry to the examination ranged from 2 to 25 years. The majority of the attending patients were Caucasian 30/32 (94%), 1/32 (3%) was Asian and 1/32 (3%) was African. Eight (25%) lived in a care home for disabled. Seven (23%) patients were employed, 4 (13%) were unemployed, 11 (35%) were students and 9 (29%) received a disability pension. One patient had Downs syndrome.

**TABLE 1:** Characteristics of the study cohort.

	Population		*p*‐Value
	Non‐attending	Attending	
Characteristics			
*N*_Total_ (male %)	158 (65)	32 (72)	0.6
Age at invitation, median (IQR)	25 (15–33)	28 (17–32)	0.6
Age at birth, GA (weeks), median (IQR)	28 (26–30)	28 (26–30)	0.7
Birth weight (g), median (IQR)	1045 (830–1440)	1033 (853–1370)	0.8
Visual impairment severity, *n* (%)			
Mild (>20/60)	7 (4)	8 (25)	0.1
Moderate (≤20/60 to >20/200)	92 (58)	14 (44)	
Severe (≤20/200 to >20/1250)	26 (17)	2 (6)	
Blind (≤20/1250 to –LP)	33 (21)	8 (25)	
Comorbidities, *n* (%)			
Combined impairments	96 (61)	14 (44)	0.07
Isolated visual impairment	62 (39)	18 (56)	

*Note*: Descriptive results are presented in median (IQR). *p*‐Value is performed with unpaired *t*‐test among attending and non‐attending regarding descriptive results. Difference in Visual impairment severity and Comorbidities were tested with Wilcoxon. Information about visual impairment severity and type of comorbidity were obtained from the NDRCVI.

The majority of patients (27/32 (84%)) had at least one neonatal complication to the preterm birth registered while they were still hospitalized at a neonatal intensive care unit (NICU), with respiratory distress syndrome, retinopathy of prematurity and periventricular haemorrhages, as the most frequent, Table 2. Retinopathy of prematurity (ROP) had not been recorded in 13 patients (41%) during neonatal screening. Nineteen patients (59%) had ROP, of whom 15 (47%) had type 1 ROP and 4 (13%) regressed spontaneously. Eight (25%) had received ROP treatment, of whom one had been treated with anti‐vascular endothelial growth factor (Lucentis/Ranibizumab) as first treatment, and seven had been treated with laser as first treatment. The earliest laser therapy within this population was performed in 2002. Seven patients (22%) born between 1988 and 1994 had been screened irregularly for ROP and all seven developed at least ROP stage 4a in between the screening visits. Vitreo‐retinal surgery was performed in two patients with reattachment of the retina, but a visual function equivalent to blindness.

**TABLE 2:** Neonatal complications registered during hospitalization at a neonatal care unit.

Neonatal diagnosis	*n* (%)
All, *n*	32 (100)
Patients with a neonatal diagnosis	27 (84)
Respiratory distress syndrome	20 (63)
Retinopathy of prematurity	19 (59)
Periventricular or intraventricular haemorrhage	14 (44)
Persistent ductus arteriosus	13 (41)
Septicemia	12 (38)
Hydrocephalus	5 (16)
Bronco‐pulmonary dysplasia (BPD)	4 (13)
Neonatal asphyxia	4 (13)
Necrotizing enterocolitis (NEC)	4 (13)
Hypoglycemia	3 (9)
Intracranial cysts	2 (6)
Periventricular leukomalacia (PVL)	1 (3)
Meningitis	1 (3)
CMV infection	1 (3)
Small for gestational age (SGA)	1 (3)
Patients without systemic disease	1 (3)
Patients with unknown neonatal history^a^	4 (13)

*Note*: More than one diagnosis can appear in the same child.

^a^ Four patients had unknown neonatal diagnosis due to birth outside Denmark or information not available in the medical records.

At the time of examination, three (9%) patients had an ocular prosthesis on one eye and two (6%) had ocular prosthesis on both eyes. A clear cornea was found in 25 (78%) patients, but band keratopathy was present in 7 (22%) (see Table 3). Further, retinal ultrastructure evaluated by optical coherence tomography (OCT) was normal in 1 (3%) patient, not evaluable in 22 (69%) and pathologic in 13 (41%) (Table 3).

**TABLE 3:** Structural characteristics at follow‐up.

	Right eyes		Left eyes		Patients
	*n* (%)	Median (IQR)	*n* (%)	Median (IQR)	*n*
Eye					
Eyes	28 (87)		29 (91)		30
Prosthesis	4 (13)		3 (9)		5
Ocular biometry					
Corneal curvature Kmean (mm)	20 (63)	7.5 (7.3–7.7)	19 (59)	7.3 (7.2–7.7)	21
Anterior chamber depth (mm)	14 (44)	3.1 (2.9–3.4)	14 (44)	3.1 (2.9–3.4)	16
Lens thickness (mm)	13 (41)	4.1 (3.7–4.4)	13 (41)	3.9 (3.8–4.1)	15
Axial length (mm)	16 (50)	23.1 (21.7–24.6)	15 (47)	22.1 (21.4–24.1)	18
Anterior segment					
Intra ocular pressure (mmHg)	25 (78)	13 (11–16)	25 (78)	13 (10–16)	28
Cornea					
Normal	24 (75)		23 (72)		25
Cornea Calcification	4 (13)		7 (22)		7
Lens					
Normal/No cataract	18 (56)		19 (59)		19
Cataract	4 (13)		4 (13)		6
IOL	2 (6)		1 (3)		3
Aphakia	1 (3)		0 (0)		1
Not evaluable	7 (22)		8 (25)		10
Posterior segment					
Retina					
Normal	1 (3)		2 (6)		2
Arrested macular development	3 (9)		3 (9)		4
Inner retinal atrophy	4 (13)		3 (9)		4
Retinal dystrophy	1 (3)		1 (3)		1
Inner retinal atrophy and arrested macular development	3 (9)		3 (9)		4
Not evaluable/Not done	20 (63)		20 (63)		22
Optic nerve					
Normal	21 (66)		22 (69)		20
Optic atrophy	6 (19)		6 (19)		6
Not evaluable/Not done	5 (16)		4 (13)		6

*Note*: The structural characteristics in 32 patients including ocular biometry, anterior‐ and posterior segment specifications. Five patients had prosthesis on at least one eye. Patients with corneal calcification was not included in the corneal curvature values.

### 3.2 Visual function

#### 3.2.1 Visual impairment severity groups

**FIGURE 2:**
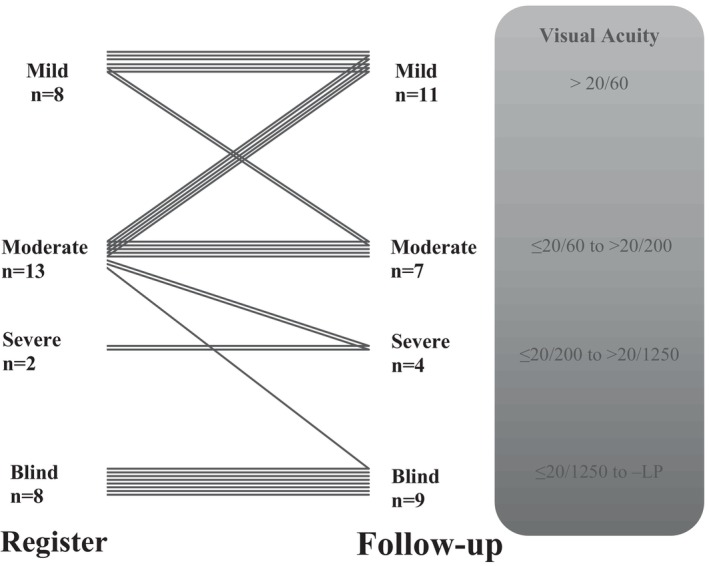
Change in grouping of visual impairment from latest entry in the registry to current examination. Each line represents one patient. Data presented for 31 patients and one patient could not corporate to the VA testing.

At the time of examination, 9 (29%) participants were blind, 4 (13%) had severe VI, 7 (23%) had a moderate VI and 11 (35%) had mild VI. The severity of VI was unchanged in most patients (*n* = 21, 68%), but five (16%) patients deteriorated by one (*n* = 4) or two (*n* = 1) levels, and 5 (16%) had improved vision compared to the latest available data in the registry (see Figure 2). One patient could not cooperate to the VA assessment.

**FIGURE 3:**
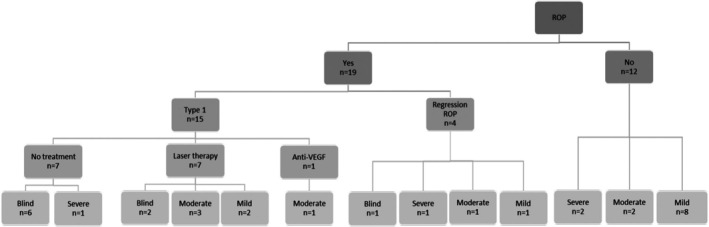
Visual outcomes at follow‐up depending on neonatal ROP status. For ROP treatment, only the first treatment is presented. VI, visual impairment. VI severity was not measured in one patient at follow‐up, this patient had no ROP. Hence, the VI severity at follow‐up are presented for 31 patients.

At follow‐up, the highest rate of blindness was observed among patients with a history of untreated ROP (6/7, 85.7%), followed by treated ROP (2/8, 25%) and lastly regressed ROP (1/4, 25%). No new cases of blindness were detected at follow‐up in patients, where ROP had not been present during hospitalization in NICU. The VI severity at follow‐up according to the neonatal ROP status and treatment is presented in Figure 3.

No patients were downgraded in VI severity group due to decreased visual field (<10° or <20°) or hemianopia on the best seeing eye. Goldmann kinetic perimetry target size lll4e could be performed on 16 patients (28 eyes). Median sum of eight meridians was 359° (IQR 339–402) and 374.1° (IQR 298–402) on right and left eyes, respectively.

#### 3.2.2 Refraction and visual aids

Nearly two‐third (*n* = 20, 63%) of the patients wore spectacles. Median spherical equivalent refraction in diopters (D) was −0.75D (IQR −8.25 to 3.25) on the right eyes and −0.88D (IQR −6.5 to 2.6) on the left eyes. Refraction was not available in 8 (25%) patients due to retinal detachment, prosthesis, phthisis or severe band keratopathy. The most used visual aid was a smartphone (12/32, (37.5%), white cane (8/32, 25%), binoculars (7/32, 21.9%), magnifiers (5/32, 15.6%), electronic aids for reading (5/32, 15.6%), closed circuit television (4/32, 12.5%) and 3 (9.4%) used a non‐verbal communication board.

### 3.3 Cause of visual impairment

**FIGURE 4:**
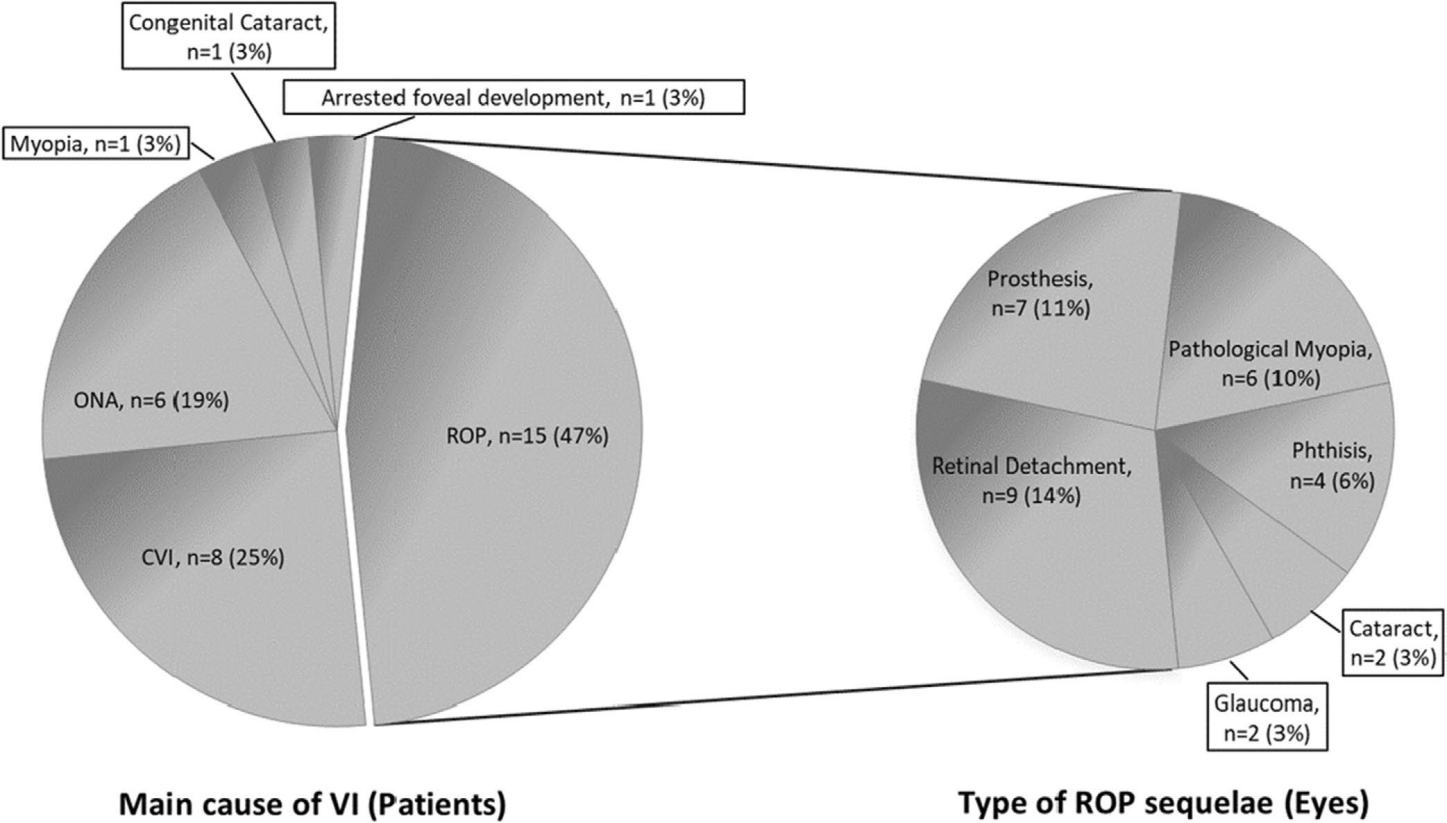
Cause of visual impairment at follow up visit. The pie (left) represents the causes of the visual impairment pr. patient evaluated at follow‐up visit. The pie (right) represents the type of ROP sequelae pr. eye.

At follow‐up, VI in 15 (47%) patients (30 eyes) could be attributed to sequelae of ROP. Among these, 9 patients (28%) had developed additional secondary conditions compared to the register data, including glaucoma, retinal detachment, uveitis, cataract or enucleation surgery. CVI was found in 8 (25%) patients, followed by optic atrophy in 6 (19%) patients, high myopia as the only identifiable cause of VI in 1 (3%) patient, one (3%) patient had arrested macular development, and finally, one (3%) patient was visually impaired due to congenital cataract (Figure 4).

### 3.4 Questionnaire

**FIGURE 5:**
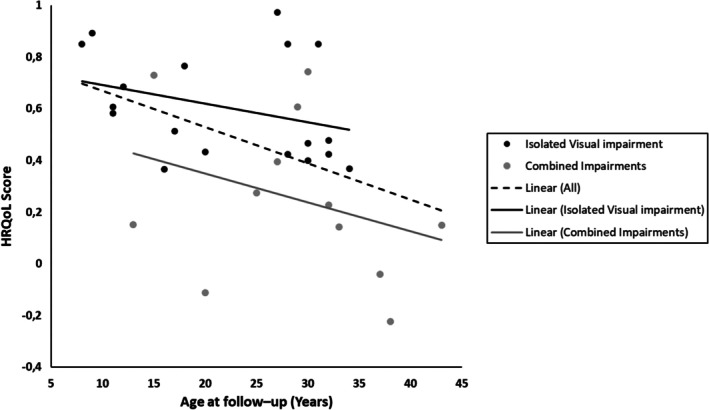
Correlation of HRQoL with age at follow‐up sorted in type of disability. A two‐way scatterplot presenting the spearman correlation coefficient between the HRQoL score assessed with the HUI3 and age at follow‐up. Showing a statistically decrease of the HRQoL with increasing age at assessment, black dashed‐line (*r* = −0.49, *p* = 0.007). The association between HRQoL and age is no longer significant when sorted in type of impairment (Isolated VI vs. combined impairment), solid line black and grey, respectively. The age was not statistically different among the two impairment types.

Twenty patients and 10 guardians filled out the HUI3 questionnaire, thus HRQoL was available for 30 patients. Most patients and guardians rated HRQoL as moderate or severe disability level (*n* = 28, 93%). The median HRQoL score was 0.45 (IQR 0.27–0.72) equivalent to severe disability level. Low HRQoL score was significantly associated with low BW (*r* = −0.4, *p* = 0.02) but not with GA (*r* = −0.18, *p* = 0.34) nor sex (*p* = 0.5). HRQoL score was negatively associated with increasing age (*r* = −0.49, *p* = 0.007). However, this association was no longer significant when sorted in type of impairment; combined impairments and isolated VI, (*r* = −0.43, *p* = 0.07 and *r* = −0.41, *p* = 0.18), respectively (Figure 5). Patients with combined impairments 12 (40%) had a significantly lower HRQoL score compared to patients with isolated VI 18 (60%), 0.19 versus 0.55, *p* = 0.003, respectively (see Table 4). The age was not statistically different among the two impairment types *p* > 0.05. A regression analysis revealed that Isolated VI is a significant predictor of HRQoL (*p* = 0.005), while age, despite showing a trend, is not statistically significant in the presence of disability (*p* = 0.1).

**TABLE 4:** HRQoL associations.

Health related quality of life (HUI3)			
	*n* (%)	HRQoL‐score, median (IQR)	*p*‐Value
HRQoL Severity Score			
None (>0.99)	0 (0)	0 (0)	
Mild (0.89–0.99)	2 (7)	0.93 (0.89–0.97)	
Moderate (0.70–0.88)	6 (20)	0.81 (0.74–0.85)	
Severe (<0.70)	22 (73)	0.40 (0.15–0.48)	
Birth parameters			
Age at follow‐up (years)		28 (17–32)	0.007
Gestational age at birth (weeks)		28 (26–30)	0.34
Birth weight (g)		1033 (853–1370)	0.02
Comorbidities, *n* = 30			
Combined impairments	12 (40)	0.19 (0.05–0.50)	0.003
Isolated visual impairment	18 (60)	0.55 (0.42–0.85)	
VI severity at follow‐up, *n* = 29			
Mild (>20/60)	11 (38)	0.58 (0.27–0.76)	0.25
Moderate (≤20/60 to >20/200)	7 (24)	0.69 (0.36–0.85)	
Severe (≤20/200 to >20/1250)	3 (10)	0.48 (−0.22–0.61)	
Blind (≤20/1250 to –LP)	8 (28)	0.41 (0.30–0.43)	
Main cause of VI, *n* = 30			
ROP	15 (47)	0.43 (0.37–0.61)	0.21
CVI	8 (25)	0.27 (0.14–0.51)	
ONA	6 (19)	0.74 (0.61–0.85)	
Myopia	1 (3)	0.04 (−0.04–0.04)	
Congenital cataract	1 (3)	0.85 (0.85–0.85)	
Arrested foveal development	1 (3)	0.97 (0.97–0.97)	
Mode of assessment, *n* = 30			
Proxy‐assessed	10 (33)	0.47 (0.15–0.69)	0.58
Self‐assessed	20 (67)	0.45 (0.38–0.73)	

*Note*: HRQoL association with severity score, comorbidities, VI severity, cause of VI and type of questionnaire assessment. *p*‐Value for birth parameters is spearman's correlation. Evaluation of difference in HRQoL‐score regarding comorbidities, VI severity, main cause and mode of assessment was tested with Kruskal–Wallis test for variables with more than two categories, otherwise *t*‐test was performed.

**FIGURE 6:**
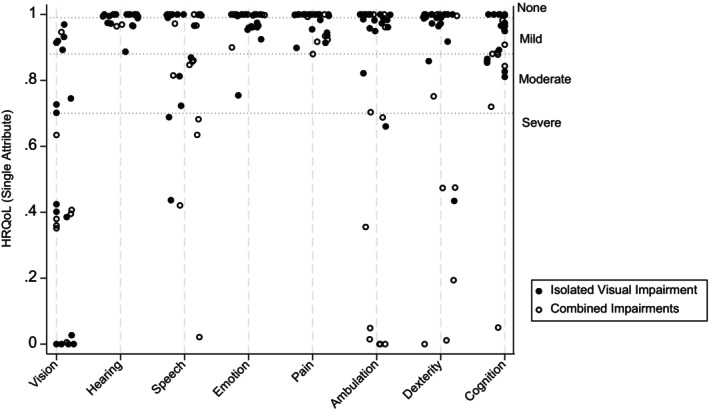
Scatterplot HRQoL score of the single attributes. HRQoL score (*y*‐axis) of each of the eight attributes (*x*‐axis), distinguishing between the patients with isolated and combined impairments. On the right *y*‐axis the severity of the score (none, mild, moderate, and severe) is marked with a dotted line. For each attribute a score for 30 patients is calculated according to the severity.

The median HRQoL was not associated with the main cause of VI (*p* = 0.21), nor with the presence (*p* = 0.3) or treatment (*p* = 0.6) of ROP, nor with the VI severity groups from the register (*p* = 0.13) or from follow‐up (*p* = 0.2). Further, no difference was detected between the mode of assessment, i.e., proxy 0.4 versus self‐assessed 0.5 (*p* = 0.58). The most affected single‐attribute utility in an ascending order, of which 0 is the worst and 1 is the best, was vision (0.5), ambulation (0.77), dexterity (0.83), speech (0.85), cognition (0.90), pain (0.98), emotion (0.98) and lastly hearing (0.99) was the least affected (Figure 6). The single attribute score for vision was significantly lower in those who had had ROP (*n* = 18, 0.38) compared to those who never had had ROP (*n* = 12, 0.68), (*p* = 0.036).

## 4 DISCUSSION

This cross‐sectional study included patients born prematurely with childhood‐onset VI. Patients were recruited from a comprehensive national registry, NDRCVI. We found that the majority of patients were visually impaired due to sequelae to ROP. The self‐rated HRQoL was poor with the worst scores for vision as a single attribute and with worse mean score in patients who had combined impairments. This study further showed that HRQoL was significantly worse in older patients and in patients with lower birth weights. However, the association with age became insignificant when dividing the patients according to combined impairments and isolated VI. No association was found between HRQoL and VI severity.

Several patients in this study were born either before or shortly after the uniform implementation of ROP screening and treatment in Denmark. Initially, treatment was consisted of cryotherapy (introduced in 1986), and later with laser ablation (introduced in 2000) (Fledelius & Kjer, 2004; Fledelius & La Cour, 2004). These patients had the worst visual and structural outcomes and their disease continued to progress compared to the latest entries in the registry. Vitreo‐retinal surgery had been performed in some of these patients, but the functional outcome was not beneficial, as presented in studies elsewhere (Özsaygili et al., 2019).

The follow‐up findings on visual function revealed that the severity of VI was stable in more than half of the cases when comparing register—data with the follow‐up examination. Improvement in VI severity at follow‐up compared to registry data may be attributed to the use of preferential looking charts and limitations in obtaining monocular VA measurements in some patients. Visual deterioration was found in one patient with a retinal dystrophy, in two patients with CVI and in two patients with combined impairments. Our findings are comparable with the final report of the CRYO‐ROP study that showed overall stable VA findings at 15‐year follow‐up, considering that severe ROP in our population was treated with laser (Palmer et al., 2005) The latest follow‐up for the ETROP study was published at the age of 9 months for the participants (Good, 2004) A high percentage of our patients (*n* = 20, 63%) were born in 1997 or earlier, representing a historical group of preterm born children. Anatomic as well as functional ocular outcomes has been especially severe for children born preterm from the 1980s to mid‐1990s, due to unsystematic screening intervals for sight threatening ROP and lack of treatment resulting in a high frequency of retinal detachments (Al‐Abaiji et al., 2024). CVI and ONA have parallel neural conditions that impair the brain's visual processing, independent of the sensory damage caused by retinal conditions like ROP. These overlapping conditions complicate the determination of the primary cause of VI, as abnormalities in any part of the visual pathway—from the retina to the brain—can significantly influence overall visual function.

The HUI3 questionnaire is a validated questionnaire that offers the unique advantage of calculating both an overall mean score for HRQoL, as well as a single score for each of the eight attributes—vision, hearing, speech, ambulation, dexterity, emotion and cognition (Feeny et al., 2002; Feng et al., 2009; Horsman et al., 2003) Worth noticing, the mean score in our study was affected negatively by BW, age at assessment and combination of VI with other impairments. Our study revealed that a higher age at assessment tends to correlate with a lower score (*p* = 0.007), in accordance with other studies using HUI (Ni et al., 2021, 2022). The decrease might be explained by a greater awareness of the life challenges among the patients, for instance when encountering challenges such as finding a job and sustaining social engagements. The significance between age and HRQoL score, however, diminished, when the patients were sorted in type of impairment. The regression analysis suggested that the HRQoL score is more strongly associated with impairment type than with age, which could indicate that factors related to combined impairments may confound the relationship between age and HRQoL. These factors could include the severity of the comorbidities, or the specific challenges associated with these, which explains that we found a lower HRQoL in patients with combined impairments compared to isolated VI, in accordance with other studies using the HUI questionnaire (Ni et al., 2021, 2022; Saigal et al., 2016).

When we analysed the mean HRQoL score according to the VI severity, we surprisingly found no statistical difference in score. Nevertheless, the difference in the HUI score is more than 0.03 among the compared groups, which is considered clinically significant (Grootendorst et al., 2000; Horsman et al., 2003) In our study, patients with a history of ROP scored significantly lower in the vision attribute compared to those without ROP. Optimizing HRQoL and visual function outcomes appears most achievable by preventing severe stages of ROP altogether.

### 4.1 Strengths and limitations

The main strength of this study is that we were able to link the examination at follow‐up with the registry data supplied with medical records for an overall picture of the long‐term outcome in former preterm born patients. The widespread age intervals from 8 to 43 enabled us to analyse the association between HRQoL and age. The insignificant associations between HRQoL and main cause of VI, VI severity and ROP status could be explained by a small sample size in this study. We had hoped to include more patients, but due to multiple disabilities besides the VI (e.g., mobility and intellectual), it required strong family resources or assistance from health care professionals to participate in the study. Hence, this study likely included patients that were less affected by the preterm birth than the non‐included. Furthermore, the study did not include specific tests to evaluate CVI, nor was brain imaging conducted on any patients as part of the study protocol. The participants had multiple disabilities and although CVI was most likely the primary cause of VI, a direct diagnosis of CVI had not been established. Nonetheless, based on current international guidelines, essential diagnostic components—such as patient history, ophthalmic examinations and optometric evaluations—were available, supporting the plausibility of diagnosing these patients with CVI.

## 5 CONCLUSIONS

ROP followed by CVI and optic atrophy were the most important factors contributing to decreased visual function in this preterm population with childhood‐onset VI. In over half of the cases, the severity of VI remained unchanged from what was registered in the NDRCVI. Combined impairments and the presence of ROP showed a negative correlation with the mean HRQoL and the vision as a single utility score, respectively. This emphasizes the importance of providing every patient with lifelong and individualized rehabilitation services and support. Therefore, this study suggests that the optimal approach to achieve improved visual function and HRQoL is to prevent severe stages of ROP and limit cerebral damage leading to combined impairments and CVI. Additional research on minimizing cerebral damage in preterm children is warranted.

## FUNDING INFORMATION

This work was supported by Fight for Sight‐ Denmark, Synoptik‐Fonden, The Danish Eye Research Foundation (Øjenfonden), Fabrikant Einar Willumsens Mindelegat, Aase og Ejnar Danielsens Fond and Dagmar Marshalls Fond.

## References

[aos17461-bib-0001] Al‐Abaiji, H.A. , Nissen, K. , Slidsborg, C. , La Cour, M. & Kessel, L. (2024) Blindness is decreasing among children born preterm during the last four decades in Denmark. Acta Ophthalmologica, 102(5), 1–8. Available from: 10.1111/aos.16625 38186309

[aos17461-bib-0002] Bolbocean, C. , Anderson, P.J. , Bartmann, P. , Cheong, J.L.Y. , Doyle, L.W. , Wolke, D. et al. (2023) Comparative evaluation of the Health Utilities Index Mark 3 and the short form 6D: evidence from an individual participant data meta‐analysis of very preterm and very low birthweight adults. Quality of Life Research, 32(6), 1703–1716. Available from: 10.1007/s11136-023-03344-x 36705795 PMC10172285

[aos17461-bib-0003] Boonstra, F.N. , Bosch, D.G.M. , Geldof, C.J.A. , Stellingwerf, C. & Porro, G. (2022) The multidisciplinary guidelines for diagnosis and referral in cerebral visual impairment. Frontiers in Human Neuroscience, 16, 1–24. Available from: 10.3389/fnhum.2022.727565 PMC9280621 35845239

[aos17461-bib-0004] Dutton, G.N. (2013) The spectrum of cerebral visual impairment as a sequel to premature birth: an overview. Documenta Ophthalmologica, 127(1), 69–78. Available from: 10.1007/s10633-013-9382-1 23657712

[aos17461-bib-0005] Feeny, D. , Furlong, W. , Torrance, G.W. , Goldsmith, C.H. , Zhu, Z. , Depauw, S. et al. (2002) Multiattribute and single‐attribute utility functions for the Health Utilities Index Mark 3 system. Medical Care, 40(2), 113–128. Available from: 10.1097/00005650-200202000-00006 11802084

[aos17461-bib-0006] Feng, Y. , Bernier, J. , McIntosh, C. & Orpana, H. (2009) Validation of disability categories derived from Health Utilities Index Mark 3 scores. Health Reports, 20(2), 43–50. 19728585

[aos17461-bib-0007] Fledelius, H.C. & Kjer, B. (2004) Surveillance for retinopathy of prematurity in a Danish country. Epidemiological experience over 20 years. Acta Ophthalmologica Scandinavica, 82(1), 38–41. Available from: 10.1046/j.1600-0420.2003.00199.x 14982044

[aos17461-bib-0008] Fledelius, H.C. & La Cour, M. (2004) Behandlingskrævende præmaturitetsretinopati i Danmark (Danish). The Journal of the Danish Medical Association, 166, 4360–4362. 15587627

[aos17461-bib-0009] Good, W.v. (2004) Final results of the early treatment for retinopathy of prematurity (ETROP) randomized trial. Transactions of the American Ophthalmological Society, 102, 233–248. 15747762 PMC1280104

[aos17461-bib-0010] Grootendorst, P. , Feeny, D. & Furlong, W. (2000) Health Utilities Index Mark 3: evidence of construct validity for stroke and arthritis in a population health survey. Medical Care, 38(3), 290–299. Available from: 10.1097/00005650-200003000-00006 10718354

[aos17461-bib-0011] Hellström, A. , Jacobson, L. , Al‐Hawasi, A. , Hellström‐Westas, L. , Rakow, A. , Johnson, M. et al. (2022) Retrospective evaluation of ophthalmological and neurological outcomes for infants born before 24 weeks gestational age in a Swedish cohort. BMJ Open, 12(8), e055567. Available from: 10.1136/bmjopen-2021-055567 PMC9353003 35922112

[aos17461-bib-0012] Holmström, G.E. , Källen, K. , Hellström, A. , Jakobsson, P.G. , Serenius, F. , Stjernqvist, K. et al. (2014) Ophthalmologic outcome at 30 months' corrected age of a prospective swedish cohort of children born before 27 weeks of gestation the extremely preterm infants in Sweden study. JAMA Ophthalmology, 132(2), 182–189. Available from: 10.1001/jamaophthalmol.2013.5812 24310059

[aos17461-bib-0013] Horsman, J. , Furlong, W. , Feeny, D. & Torrance, G. (2003) The Health Utilities Index (HUI®): concepts, measurement properties and applications. Health and Quality of Life Outcomes, 1, 1–13. Available from: 10.1186/1477-7525-1-54 14613568 PMC293474

[aos17461-bib-0014] ICROP . (2021) International classification of retinopathy of prematurity, third edition. Ophthalmology, 128(10), e51–e68. Available from: 10.1016/j.ophtha.2021.05.031 34247850 PMC10979521

[aos17461-bib-0032] Jain, S. , Sim, P. Y. , Beckmann, J. , Ni, Y. , Uddin, N. , Unwin, B. et al. (2022) Functional Ophthalmic Factors Associated With Extreme Prematurity in Young Adults. JAMA Network Open, 5(1), e2145702. Available from: 10.1001/jamanetworkopen.2021.45702 35089350 PMC8800073

[aos17461-bib-0015] Kessel, L. , Jensen, H. , Larsen, A. , Rosenberg, T. & Nissen, K.R. (2024) Temporal changes in incidence, prevalence and causes of childhood visual impairment – learnings from 45 years with the National Danish Registry of Children with Visual Impairment. Acta Ophthalmologica, 102(7), 790–796. Available from: 10.1111/aos.16700 38662528

[aos17461-bib-0016] Larsson, E. , Martin, L. & Holmström, G. (2004) Peripheral and central visual fields in 11‐year‐old children who had been born prematurely and at term. Journal of Pediatric Ophthalmology & Strabismus, 41, 39–45. 14974834 10.3928/0191-3913-20040101-10

[aos17461-bib-0017] McLoone, E. , O'Keefe, M. , McLoone, S. & Lanigan, B. (2007) Effect of diode laser retinal ablative therapy for threshold retinopathy of prematurity on the visual field: results of goldmann perimetry at a mean age of 11 years. Journal of Pediatric Ophthalmology and Strabismus, 44(3), 170–173. 17542439 10.3928/0191-3913-20070301-10

[aos17461-bib-0018] Merabet, L.B. , Mayer, L. , Bauer, C.M. , Wright, D. & Kran, B.S. (2017) Disentangling how the brain is “wired” in cortical/cerebral visual impairment (CVI). Seminars in Pediatric Neurology, 24(2), 83–91. Available from: 10.1016/j.spen.2017.04.005.Disentangling 28941531 PMC5659284

[aos17461-bib-0019] Mitha, A. , Chen, R. , Razaz, N. , Johansson, S. , Stephansson, O. , Altman, M. et al. (2024) Neurological development in children born moderately or late preterm: national cohort study. BMJ: British Medical Journal, 384, e075630. Available from: 10.1136/bmj-2023-075630 38267070 PMC11957549

[aos17461-bib-0020] Ni, Y. , Johnson, S. , Marlow, N. & Wolke, D. (2022) Reduced health‐related quality of life in children born extremely preterm in 2006 compared with 1995: the EPICure studies. Archives of Disease in Childhood. Fetal and Neonatal Edition, 107(4), 408–413. Available from: 10.1136/archdischild-2021-322888 34697040 PMC9209681

[aos17461-bib-0021] Ni, Y. , O'Reilly, H. , Johnson, S. , Marlow, N. & Wolke, D. (2021) Health‐related quality of life from adolescence to adulthood following extremely preterm birth. Journal of Pediatrics, 237, 227–236. Available from: 10.1016/j.jpeds.2021.04.005 33836186

[aos17461-bib-0022] Özsaygili, C. , Ozdek, S. , Ozmen, M.C. , Atalay, H.T. & Yalinbas Yeter, D. (2019) Parameters affecting postoperative success of surgery for stage 4A/4B ROP. British Journal of Ophthalmology, 103(11), 1624–1632. Available from: 10.1136/bjophthalmol-2018-312922 30658990

[aos17461-bib-0023] Palmer, E.A. , Hardy, R.J. , Dobson, V. , Phelps, D.L. , Quinn, G.E. , Summers, C.G. et al. (2005) 15‐year outcomes following threshold retinopathy of prematurity: final results from the multicenter trial of cryotherapy for retinopathy of prematurity. Archives of Ophthalmology, 123(3), 311–318. Available from: 10.1001/archopht.123.3.311 15767472

[aos17461-bib-0024] Pascal, A. , Govaert, P. , Oostra, A. , Naulaers, G. , Ortibus, E. & Van den Broeck, C. (2018) Neurodevelopmental outcome in very preterm and very‐low‐birthweight infants born over the past decade: a meta‐analytic review. Developmental Medicine and Child Neurology, 60(4), 342–355. Available from: 10.1111/dmcn.13675 29350401

[aos17461-bib-0025] Ruberto, G. , Salati, R. , Milano, G. , Bertone, C. , Tinelli, C. , Fazzi, E. et al. (2006) Changes in the optic disc excavation of children affected by cerebral visual impairment: a tomographic analysis. Investigative Opthalmology & Visual Science, 47(2), 484–488. Available from: 10.1167/iovs.05-0529 16431940

[aos17461-bib-0026] Saigal, S. , Ferro, M.A. , Van Lieshout, R.J. , Schmidt, L.A. , Morrison, K.M. & Boyle, M.H. (2016) Health‐related quality of life trajectories of extremely low birth weight survivors into adulthood. Journal of Pediatrics, 179, 68–73.e1. Available from: 10.1016/j.jpeds.2016.08.018 27592095

[aos17461-bib-0027] Sakki, H. , Bowman, R. , Sargent, J. , Kukadia, R. & Dale, N. (2021) Visual function subtyping in children with early‐onset cerebral visual impairment. Developmental Medicine and Child Neurology, 63(3), 303–312. Available from: 10.1111/dmcn.14710 33111315

[aos17461-bib-0028] Sakki, H. , Dale, N.J. , Sargent, J. , Perez‐Roche, T. & Bowman, R. (2018) Is there consensus in defining childhood cerebral visual impairment? A systematic review of terminology and definitions. British Journal of Ophthalmology, 102(4), 424–432. Available from: 10.1136/bjophthalmol-2017-310694 29146757

[aos17461-bib-0029] Sharma, S. , Chitranshi, N. , Wall, R. , Vander Basavarajappa, D. , Gupta, V. , Mirzaei, M. et al. (2022) Trans‐synaptic degeneration in the visual pathway: neural connectivity, pathophysiology, and clinical implications in neurodegenerative disorders. Survey of Ophthalmology, 67(2), 411–426. Available from: 10.1016/j.survophthal.2021.06.001 34146577

[aos17461-bib-0030] Slidsborg, C. , Bangsgaard, R. , Fledelius, H.C. , Jensen, H. , Greisen, G. & La Cour, M. (2012) Cerebral damage may be the primary risk factor for visual impairment in preschool children born extremely premature. Archives of Ophthalmology, 130(11), 1410–1417. Available from: 10.1001/archophthalmol.2012.1393 22688255

[aos17461-bib-0031] Sveinsdóttir, K. , Ley, D. , Hövel, H. , Fellman, V. , Hüppi, P.S. , Smith, L.E.H. et al. (2018) Relation of retinopathy of prematurity to brain volumes at term equivalent age and developmental outcome at 2 years of corrected age in very preterm infants. Neonatology, 114(1), 46–52. Available from: 10.1159/000487847 29649829 PMC5997524

